# VEGFR2 Expression Correlates with Postnatal Development of Brain Arteriovenous Malformations in a Mouse Model of Type I Hereditary Hemorrhagic Telangiectasia

**DOI:** 10.3390/biomedicines11123153

**Published:** 2023-11-27

**Authors:** Chul Han, Candice L. Nguyen, Lea Scherschinski, Tyler D. Schriber, Helen M. Arthur, Michael T. Lawton, Suk Paul Oh

**Affiliations:** 1 Barrow Aneurysm and AVM Research Center, Department of Translational Neuroscience, Barrow Neurological Institute, St. Joseph’s Hospital and Medical Center, Phoenix, AZ 85013, USA; chul.han@barrowneuro.org (C.H.); candice.nguyen@louisville.edu (C.L.N.); lea.scherschinski@barrowneuro.org (L.S.); michael.lawton@barrowbrainandspine.com (M.T.L.); 2 Department of Neurosurgery, Barrow Neurological Institute, St. Joseph’s Hospital and Medical Center, Phoenix, AZ 85013, USA; 3 Department of Neurosurgery, Charité—Universitätsmedizin Berlin, Corporate Member of Freie Universität Berlin, Humboldt-Universität zu Berlin, and Berlin Institute of Health, 10117 Berlin, Germany; 4 Biosciences Institute, Newcastle University, Newcastle NE1 7RU, UK; helen.arthur@newcastle.ac.uk

**Keywords:** endoglin, brain arteriovenous malformation, hereditary hemorrhagic telangiectasia, angiogenesis, vascular disorder, magnetic resonance angiography

## Abstract

Brain arteriovenous malformations (BAVMs) are a critical concern in hereditary hemorrhagic telangiectasia (HHT) patients, carrying the risk of life-threatening intracranial hemorrhage. While traditionally seen as congenital, the debate continues due to documented *de novo* cases. Our primary goal was to identify the precise postnatal window in which deletion of the HHT gene Endoglin (*Eng*) triggers BAVM development. We employed *Scl*CreER(+);*Eng*^2f/2f^ mice, enabling timed *Eng* gene deletion in endothelial cells via tamoxifen. Tamoxifen was given during four postnatal periods: P1–3, P8–10, P15–17, and P22–24. BAVM development was assessed at 2–3 months using latex dye perfusion. We examined the angiogenic activity by assessing vascular endothelial growth factor receptor 2 (VEGFR2) expression via Western blotting and *Flk1*-LacZ reporter mice. Longitudinal magnetic resonance angiography (MRA) was conducted up to 9 months. BAVMs emerged in 88% (P1–3), 86% (P8–10), and 55% (P15–17) of cases, with varying localization. Notably, the P22–24 group did not develop BAVMs but exhibited skin AVMs. VEGFR2 expression peaked in the initial 2 postnatal weeks, coinciding with BAVM onset. These findings support the “second hit” theory, highlighting the role of early postnatal angiogenesis in initiating BAVM development in HHT type I mice.

## 1. Introduction

Arteriovenous malformations (AVMs) refer to abnormal connections between arteries and veins through a tangled low-resistance, high-flow vascular nidus that is devoid of intervening capillaries. The rupture of brain AVMs (BAVMs) results in an intracerebral hemorrhage, which is a life-threatening form of stroke. Traditionally, BAVMs have been regarded as congenital lesions present from birth. However, recent cases of *de novo* BAVMs challenge the established notion that BAVMs originate congenitally [1,2,3,4,5,6,7,8].

Approximately 95% of BAVMs are considered sporadic, lacking discernible inherited patterns. Somatic mosaic mutations in genes implicated in the rat sarcoma (RAS)-signaling pathway were identified within endothelial cells of sporadic BAVM lesions. Notably, mutations in the Kirsten RAS (*KRAS*) gene are prevalent in over 50% of human sporadic BAVMs [9,10,11].

In contrast, approximately 5% of BAVMs are familial and primarily associated with hereditary hemorrhagic telangiectasia (HHT). HHT is an autosomal-dominant vascular disease characterized by the occurrence of AVMs in the brain, lungs, and visceral organs. Mutations in the endoglin (*ENG*), activin receptor-like kinase 1 (*ACVRL1* or *ALK1*), or *SMAD4* genes were identified as the underlying cause of this disorder [12,13,14]. The proteins encoded by HHT-associated genes are crucial mediators in the signaling pathway of the bone morphogenetic protein (BMP) family of growth factors. Recent genetic studies provided evidence that the development of AVMs is underpinned by a deficiency in the linear ENG-ALK1-SMAD4 pathway [15,16,17]. In mouse models of HHT, the induction of AVMs requires heterozygous [15,18] or homozygous deletion of the *Alk1*, *Eng*, or *Smad4* gene [16,17,19]. Furthermore, additional factors such as wounding or angiogenic stimulation, in addition to the genetic deletion of HHT genes, are necessary for inducing AVMs in adult mice [17,20,21]. In mouse models of familial BAVMs, the deletion of HHT-associated genes during embryonic and neonatal stages resulted in the concurrent development of BAVMs and visceral AVMs [17,22,23,24]. However, when the *Alk1* or *Eng* gene was deleted during adulthood, it led to the formation of visceral AVMs but not BAVMs [25]. Nevertheless, viral vector-mediated delivery of vascular endothelial growth factor (VEGF), which is the most potent angiogenic factor, along with genetic deletions of *Eng* or *Alk1*, resulted in BAVM-like cerebrovascular dysplasia in adult mice [21,22,26]. This suggests that HHT-associated gene deletion alone is insufficient to induce BAVMs in adult mice, emphasizing the need for additional angiogenic stimulation for BAVM development in adulthood. In contrast, a recent study with sporadic BAVM models revealed that endothelial-cell-specific induction of mutant KRAS expression alone was sufficient for BAVM development in adult mice [27,28]. Collectively, these observations suggest the possibility of distinct underlying mechanisms between familial and sporadic cases in *de novo* development of BAVMs.

In this study, we investigated the specific postnatal stages at which the deletion of an HHT-associated gene triggers the development of BAVMs. To identify these critical stages, we employed endothelial-cell-specific, tamoxifen-inducible conditional *Eng* mutant mice. Our investigations revealed that BAVMs exclusively formed when *Eng* was deleted within the first 2 weeks of the postnatal period. This timeframe was closely aligned with heightened VEGFR2 expression levels in the brain. These results suggest that a proangiogenic milieu during postnatal brain development may be critical for the development of BAVMs in this type I HHT mouse model. Furthermore, most *Eng* mutants displayed BAVMs in the forebrain and hindbrain, thus rendering this model a useful preclinical model for studying forebrain and cerebellar BAVMs.

## 2. Materials and Methods

### 2.1. Transgenic Mice and Conditional Gene Deletion

All animal procedures were conducted in accordance with guidelines established by the Institutional Animal Care and Use Committee at Barrow Neurological Institute and St. Joseph Hospital Medical Center (protocol #573). The *Eng*^2f^ alleles were established in laboratory mice using techniques previously described [29]. The inducible endothelial-cell-specific Cre transgenic mouse line, namely, *Scl*CreER(+) [30], was generously provided by Dr. Yunchao Su. While the conventional knockout of the *Eng* gene results in early embryonic lethality [15,31], the conditional knockout in *Scl*CreER(+) mice allows for the postnatal time- and tissue-specific deletion of the *Eng* gene, particularly in the endothelium [20]. To generate mutant mice (*Scl*CreER(+);*Eng*^2f/2f^), we crossed *Scl*CreER(+) mice with floxed conditional *Eng* deletion mice (*Eng*^2f/2f^). These mice were on a mixed (129Sv/C57BL6) background. The *Eng* gene was deleted at 4 different stages of postnatal life by administering tamoxifen (50 μg/day; Sigma-Aldrich, St. Louis, MO, USA) intragastrically for 3 consecutive days at postnatal days P1–3, P8–10, P15–17, or P22–24. An alternative high-dose tamoxifen strategy that used a dosage of 250 μg/day was administered for 3 consecutive days at P22–24. We employed CreER-negative *Eng*^2f/2f^ mice treated with tamoxifen as controls for each group. Hereafter in the manuscript, the term “control” indicates CreER-negative *Eng*^2f/2f^ mice treated with tamoxifen. Furthermore, we utilized *Flk1*-LacZ knock-in reporter mice, which were kindly provided by Dr. J. Rossant, in a mixed (129Sv/C57BL6) background to analyze the promoter activity of *Flk1*, which encodes vascular endothelial growth factor receptor 2 (VEGFR2).

### 2.2. AVM Visualization Using Latex Dye Perfusion

Mice were anesthetized using a ketamine (100 mg/kg body weight; ) and xylazine (10 mg/kg body weight) mixture. Following this, the thoracic cavity was opened to expose the heart. Blue latex dye (5 μL/g body weight; VWR, Radnor, PA, USA) was injected through the left heart following sequential perfusion with vasodilating and fixative reagents, as previously described [32]. After overnight fixation, the brains were isolated, dehydrated, and cleared using organic solvents in accordance with established methods [17]. The cleared brains were sectioned into 1 mm thick coronal slices using a brain slicer matrix (Zivic Instruments, Pittsburgh, PA, USA). The sectioned brains were imaged with a CCD camera (Leica, Allendale, NJ, USA) to capture the latex-dye-perfused cerebrovasculature and BAVMs. To assess the formation of skin AVMs, a wound was induced at 3 weeks of age through ear tagging. This was followed by the perfusion of 0.5 mL latex dye through the left heart at 2 to 3 months of age. After overnight fixation, the ears were placed on Styrofoam and cleared using organic solvents by following the previously described protocol [17].

### 2.3. Hemoglobin Concentration

Hemoglobin levels in the blood were quantified using a hemoglobin photometer (Hemopoint H2, STANBIO Laboratory, Boerne, TX, USA).

### 2.4. Western Blotting

The right cerebrum brain tissues from CreER-negative *Eng*^2f/2f^ mice without tamoxifen treatment at various postnatal stages (P8, P15, P22, and P29) were lysed using a RIPA lysis buffer (Thermo Fisher Scientific, Waltham, MA, USA) containing 1× Halt™ Protease Inhibitor Cocktail (Pierce) and 1X Halt™ Phosphatase Inhibitor Cocktail (Pierce). The protein concentration was determined using the DC™ Protein Assay kit (Bio-Rad Laboratories, Hercules, CA, USA), and proteins were separated using SDS-PAGE. Subsequently, the samples were transferred onto a nitrocellulose membrane (Bio-Rad Laboratories, Hercules, CA, USA). The following primary antibodies were used: anti-VEGFR2 (2479, Cell Signaling Technology, Danvers, MA, USA; 1:1000), anti-CD31 (ab124432, Abcam, Waltham, MA, USA; 1:1000), and anti-β-actin (A1978, Sigma-Aldrich, St. Louis, MO, USA; 1:5000). The secondary antibodies included HRP-conjugated anti-rabbit IgG (NA934, GE healthcare, Chicago, IL, USA; 1:5000) and HRP-conjugated anti-mouse IgG (NA931, GE healthcare, Chicago, IL, USA; 1:5000). Detection was accomplished using the ECL Western blotting substrate (Thermo Fisher Scientific, Waltham, MA, USA), and the bands were quantified using ImageJ software, version 1.53t.

### 2.5. Magnetic Resonance Imaging

BAVMs in mice were examined using brain magnetic resonance imaging (MRI) and 3D time-of-flight magnetic resonance angiography (MRA). Each mouse was induced and maintained under isoflurane anesthesia (3% induction, 1–2% maintenance) in medical air. Respiratory activity was continuously monitored using a pillow sensor positioned under the abdomen (SA Instruments, Stony Brook, NY, USA), and normal body temperature (36–37 °C) was maintained using a circulating warm water blanket (Thermo Fisher Scientific, Rockford, IL, USA).

MRI was performed using a 7 T small-animal, 30 cm horizontal-bore magnet and BioSpec Avance III spectrometer (Bruker, Billerica, MA, USA) with a 116 mm high-power gradient set (600 mT/m) and a 30 mm whole-body mouse quadrature coil. Fast spin-echo scout images were acquired in three orthogonal planes to cover the brain (repetition time (TR) = 1000 ms, echo time (TE) = 12.5 ms, effective echo time (TEeff) = 50 ms, 128 × 128 matrix, 0.234 × 0.234 × 1.5 mm voxels, number of acquisitions (NEX) = 1). These scout images were then used to determine the placement of the T2-weighted MRI and MRA images. Coronal T2-weighted rapid acquisition with relaxation enhancement MRI images were acquired to span the volume of the brain and identify BAVM lesions (TR = 5000 ms, TE = 12 ms, TEeff = 60 ms, rare factor = 8, 180 × 180 matrix, 0.1 × 0.1 × 0.5 mm voxels, 30 slices, NEX = 4). Three-dimensional time-of-flight MRA images were acquired using a flow-compensated gradient echo method (TR = 30 ms, TE = 3.5 ms, α = 30°, 180 × 180 × 200 matrix, 0.1 × 0.1 × 0.1 voxels, NEX = 4). Magnetization transfer pulses were added to the MRA acquisition to provide additional background tissue suppression (offset frequency = 1500 Hz, r.f. amplitude= 0.5 μT).

### 2.6. X-gal Staining and Vascular Density Quantification

Anesthesia was induced in *Flk1*-LacZ mice through intraperitoneal injection of a mixture comprising ketamine (100 mg/kg body weight) and xylazine (10 mg/kg body weight). Mice were transcardially perfused using a GP1000 peristaltic pump (Thermo Fisher Scientific, Waltham, MA, USA) with phosphate-buffered saline (PBS) containing heparin (50 units/mL). Brains were extracted from the cranium and fixed in a PBS solution containing 1% formaldehyde, 2 mM MgCl_2_, 5 mM EGTA, and 0.02% NP-40 for 5 min. This was followed by an additional 10 min fixation period after coronal slicing. After washing with PBS, the fixed brain sections were incubated with X-gal staining solution containing 5 mM K3Fe(CN)6, 5 mM K4Fe(CN)6, 2 mM MgCl2, 0.01% sodium deoxycholate, 0.02% NP-40, 0.5 mg/mL X-gal in dimethylformamide, PBS (1/10 vol), and 2.5 mM EGTA at 37 °C overnight. VEGFR2 (*Flk1*)-expressing blood vessels appeared blue and were imaged using a CCD camera (Leica, Allendale, NJ, USA). The vascular density was quantified using ImageJ (version 1.54a, NIH, Bethesda, MD, USA) in conjunction with the vessel analysis plugin.

### 2.7. Statistical Analysis

Categorical data are represented as numbers and percentages, and continuous data are represented as the mean and standard deviation (SD). The variations in VEGFR2/CD31/β-actin levels, the mean vascular density, and the hemoglobin levels were analyzed using one-way ANOVA with post hoc Tukey’s multiple comparison tests. The threshold for statistical significance was established as *p* < 0.05.

## 3. Results

### 3.1. Endothelial Cell Eng Gene Deletion Induced BAVM Development

To induce the deletion of the *Eng* gene within endothelial cells, we intragastrically administered tamoxifen (25 μg/g body weight) to *Scl*CreER(+);*Eng*^2f/2f^ mice for 3 consecutive days, starting on P1 (P1–3). Approximately 60% of *Scl*CreER(+);*Eng*^2f/2f^ mice treated with tamoxifen at P1–3 did not survive beyond 6 months, which was likely due to hemorrhages caused by AVMs in the gastrointestinal tract, lungs, and brain (Supplementary Figure S1A) [23,24,33]. Supporting this, those *Eng* mutants where tamoxifen was injected at P1–3 displayed lower hemoglobin levels compared with the control group (Supplementary Figure S1B).

**Figure 1:**
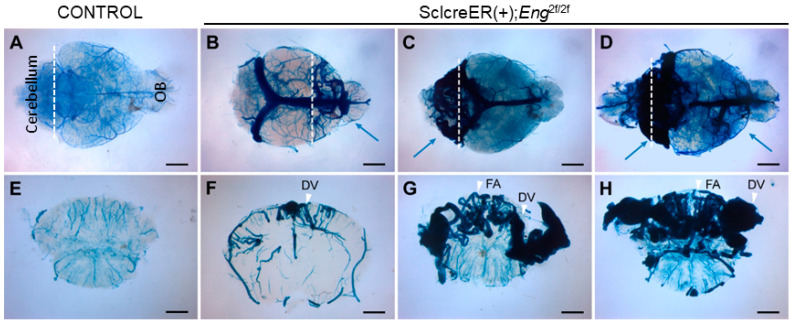
Endothelial cell deletion of the *Eng* gene at P1–3 induced BAVMs. The cerebrovasculature was visualized at age 2–3 months using latex dye perfusion and examined in whole brains (**A**–**D**) and their corresponding coronal brain sections (**E**–**H**). BAVMs were not detected in control brains (**A**,**E**), while mutant mice exhibited BAVMs in the forebrain (**B**,**F**), hindbrain (**C**,**G**), or both regions (**D**,**H**). Blue arrows indicate BAVM lesions and white arrowheads indicate the feeding arteries (FAs) and draining veins (DVs). White dashed lines indicate the cutting planes. OB: olfactory bulb. Scale bars: 2 mm.

The presence of BAVMs was assessed at 2 to 3 months of age using latex dye perfusion, which is a technique that enables the visualization of arteriovenous shunts because the latex dye passes through the nidus and directly enters enlarged venous channels (Figure 1) [33]. Approximately 90% of mutant mice treated with tamoxifen at P1–3 developed BAVMs, with a predominant localization in the forebrain (Figure 1B), cerebellum (Figure 1C), or both regions (Figure 1D). Notably, cerebellar BAVMs constituted the most frequent subtype, accounting for 48% (N = 10/21) of all BAVMs observed, followed by forebrain BAVMs (33%, N = 7/21). The BAVM phenotype was characterized by a vascular nidus composed of enlarged, tortuous vessels derived from feeding arteries and draining veins. These characteristics were discernible in latex-dye-perfused coronal brain sections (Figure 1F–H).

### 3.2. Longitudinal Monitoring of BAVMs Using Magnetic Resonance Angiography

In our previous study, we employed high-resolution MRA to study BAVM progression in *Tagln*Cre(+);*Alk1*^2f/2f^ mutant mice, where Cre recombinase was expressed in smooth muscle cells and a subset of endothelial cells [33]. In that model, BAVMs predominantly developed in the parietal lobe, establishing a reliable preclinical longitudinal model for studying brain BAVMs. Since *Scl*CreER(+);*Eng*^2f/2f^ mutant mice in this study displayed nidal BAVMs in the forebrain and cerebellum, we explored their potential as an alternative longitudinal mouse model, particularly for forebrain and cerebellar BAVMs.

**Figure 2:**
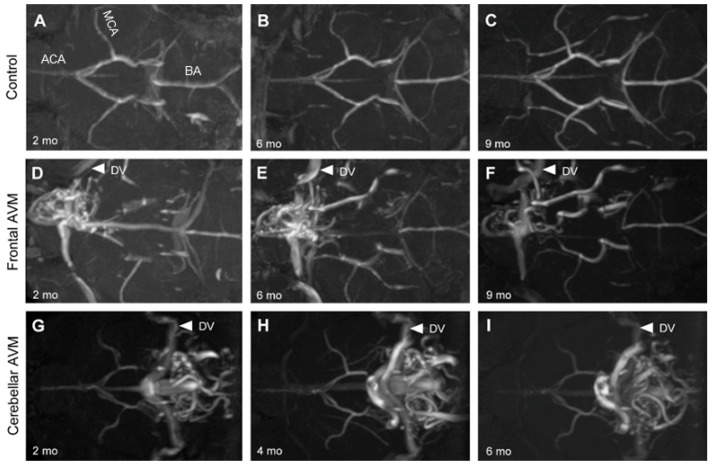
Longitudinal magnetic resonance angiography (MRA) imaging of nidal BAVMs. Endothelial cell deletion of the *Eng* gene at P1–3 induced BAVMs. BAVMs of *Eng* mutants were monitored monthly through MRA imaging. Representative MRA images show axial views of a control brain (**A**–**C**) displaying the major cerebral arteries without aberrant vasculature at 2, 6, and 9 months. Representative MRA images show axial views of a forebrain BAVM (**D**–**F**) and cerebellar BAVM (**G**–**I**) at 2, 6, and 9 months. White arrowheads indicate the draining veins (DVs). ACA: anterior cerebral artery, MCA: middle cerebral artery, BA: basilar artery.

As anticipated, the control mice displayed a normal arterial angioarchitecture of the anterior and posterior circulations, as well as the circle of Willis. No vascular malformations were observed in the control mice (*n* = 3) throughout the 9-month study duration (Figure 2A–C). In contrast, *Eng* mutant mice (81%, 17/21) exhibited forebrain and cerebellar BAVMs characterized by a vascular nidus, feeding arteries, and draining veins (Figure 2D–I). These BAVMs remained stable throughout the 9-month study period. Additionally, cerebellar BAVMs were characterized by a substantial nidus that occupied most of the cerebellum (Figure 1C,D) and bilaterally drained through the transverse sinus (Figure 2G–I).

### 3.3. The Early Postnatal Period Was Critical for BAVM Development

**Figure 3:**
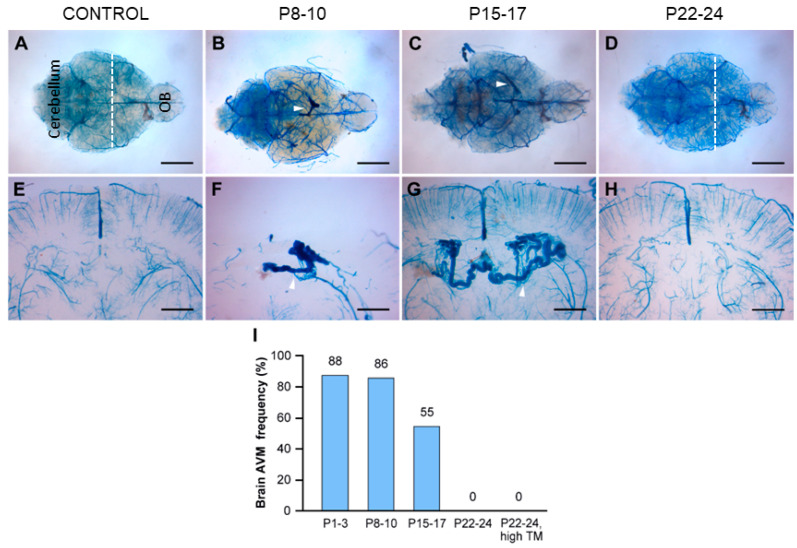
Timing of Endoglin (*Eng*) gene deletion dictated BAVM development. Tamoxifen was administered at different time points during the postnatal stage (P8–10, P15–17, P22–24; high or low dose). The cerebrovasculature was visualized using latex dye perfusion at 2–3 months of age and examined in whole brains (**A**–**D**) and their corresponding coronal brain sections (**E**–**H**). BAVMs were not detected in control brains (**A**), while *Eng* mutant mice displayed parietal lobe BAVMs in the P8–10 group (**B**) and the P15–17 group (**C**), but not in the P22–24 group (**D**). Magnified views of coronal brain sections show a normal cerebrovasculature in the control brain (**E**) and dilated, fistula-like vessels in mutant brains (**F**–**H**). White arrowheads indicate BAVM lesions in whole-brain images and enlarged, tortuous blood vessels in coronal brain sections. White dashed lines indicate the cutting planes. OB: olfactory bulb. (**I**) The bar graph shows the frequencies of postnatal BAVM development in the type I HHT mouse model among treatment groups. P1–3 (88%, *n* = 21/24), P8–10 (86%, *n* = 6/7), P15–17 (55%, *n* = 6/11), P22–24 (low dose, 0%, *n* = 0/8), and P22–24 (high dose, 0%, *n* = 0/6). Scale bars: 3 mm (**A**–**D**) and 1 mm (**E**–**H**). TM: tamoxifen.

To address the prevailing belief that BAVMs are congenital in nature, we employed a time-dependent gene deletion strategy. Our objective was to investigate the possibility of inducing BAVMs at specific postnatal time points through targeted deletion of the *Eng* gene. Tamoxifen was administered to *Eng* mutant mice at various postnatal intervals, including at 1 week (P8–10), 2 weeks (P15–17), and 3 weeks (P22–24) of age, in addition to the previously studied P1–3 timeframe. Subsequently, we assessed the presence of BAVMs in these mice at the age of 2 to 3 months using latex dye perfusion (Figure 3).

**Figure 4:**
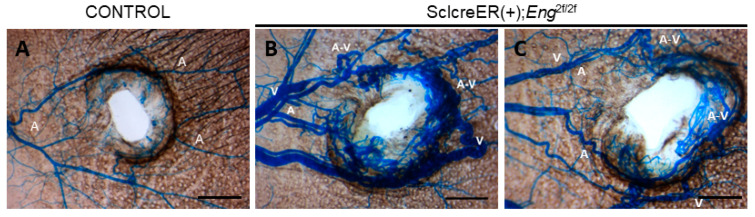
Wound-induced skin AVMs developed at 3 weeks of age in *Eng* mutants. Conditional *Eng* mutant mice (*n* = 8) and control mice (*n* = 3) were treated with high-dose tamoxifen at 3 weeks of age and wounds were induced using ear tags. Control mice displayed a high density of vessels around wounded ears but did not develop AVMs (**A**), while mutant mice developed skin AVMs around wounded ears but did not develop BAVMs (**B**,**C**). A: artery, V: vein, A-V: arteriovenous shunt. Scale bar: 1 mm.

The P8–10 group exhibited a similar frequency of BAVM formation (86%, *n* = 6/7) compared with the P1–3 group (88%, *n* = 21/24). The P15–17 group displayed a slightly lower frequency of BAVM formation (55%, *n* = 6/11) compared with the P1–3 and P8–10 groups (Figure 3I). Additionally, both the P8–10 and P15–17 groups developed smaller BAVMs compared with the P1–3 group. Notably, the majority of BAVMs in the P8–10 and P15–17 groups were localized in the parietal lobe (83%, *n* = 10/12), distinguishing them from the forebrain and cerebellar BAVMs observed in the P1–3 group (Figure 3B,C,F,G). Interestingly, the P22–24 group did not exhibit any BAVM development, raising concerns about inadequate tamoxifen levels for initiating BAVM formation, considering their increased body weight. To eliminate the possibility of insufficient tamoxifen blood levels in the P22–24 group, we increased the tamoxifen dosage (250 μg/day, 25 μg/g body weight) to match that of the P1–3 group. Nonetheless, the high-dose treatment did not yield BAVMs in the P22–24 group (Figure 3I). However, we observed skin AVMs in wounded ears (Figure 4B,C), confirming that the tamoxifen levels were adequate in the P22–24 group. Collectively, these findings suggest that within the first 2 weeks of the postnatal period, physiologic stimuli play a pivotal role in shaping the microenvironment essential for the induction of BAVMs, in conjunction with a genetically induced *Eng* deficiency.

### 3.4. VEGFR2 Expression Predominated in the Early Postnatal Period

In our quest to understand the nature of these critical physiological stimuli within the microenvironment, we focused our investigations on the proangiogenic conditions present in the early postnatal brain. This direction was informed by previous studies that demonstrated the need for angiogenic stimulation to induce BAVMs, in addition to genetic deletions of the *Alk1* or *Eng* gene [22,34]. Moreover, studies provided insights into the temporal dynamics of angiogenic activity in the rodent brain, with peak levels observed during the first postnatal week, followed by a gradual decline over the subsequent weeks [35,36,37]. Among the proangiogenic pathways, the vascular endothelial growth factor (VEGF) signaling cascade, with VEGFR2 as the primary receptor, is one of the most extensively studied [38]. Previous reports have shown that VEGFR2 expression is most pronounced during the initial 2 postnatal weeks, after which it significantly declines in the mouse brain vasculature [39].

**Figure 5:**
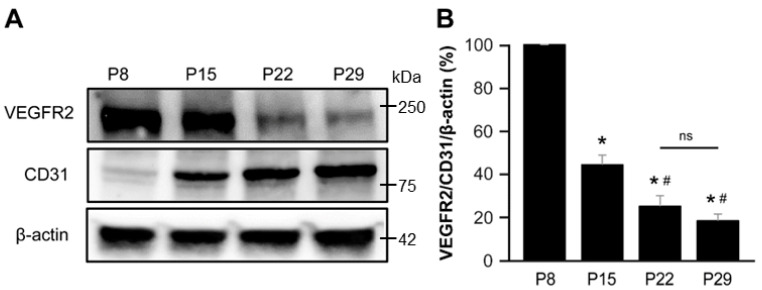
VEGFR2 was predominantly expressed during the first 2 weeks of the postnatal period in the mouse brain. (**A**) Western blot analysis results depict the protein levels of VEGFR2, CD31, and β-actin in mouse brains at P8, P15, P22, and P29. (**B**) The bar graph represents the percentage expressions of VEGFR2/CD31/β-actin at P8, P15, P22, and P29 normalized to the P8 levels. The P8 and P15 groups showed significantly higher expressions of VEGFR2 compared with the P22 and P29 groups. *, *p* < 0.001 vs. P8; #, *p* < 0.005 vs. P15; N.S., not significant. *n* = 5.

To substantiate these findings, we analyzed the protein expression of VEGFR2 and CD31 in the brains of normal mice (Cre-negative, *Eng*^2f/2f^ mice) at various postnatal time points, including P8, P15, P22, and P29. Our analysis revealed that VEGFR2 expression in the brain was notably abundant during the initial 2-week postnatal period and gradually declined thereafter. Conversely, the expression of CD31, which is an endothelial cell marker, continued to increase throughout the postnatal period (Figure 5).

**Figure 6:**
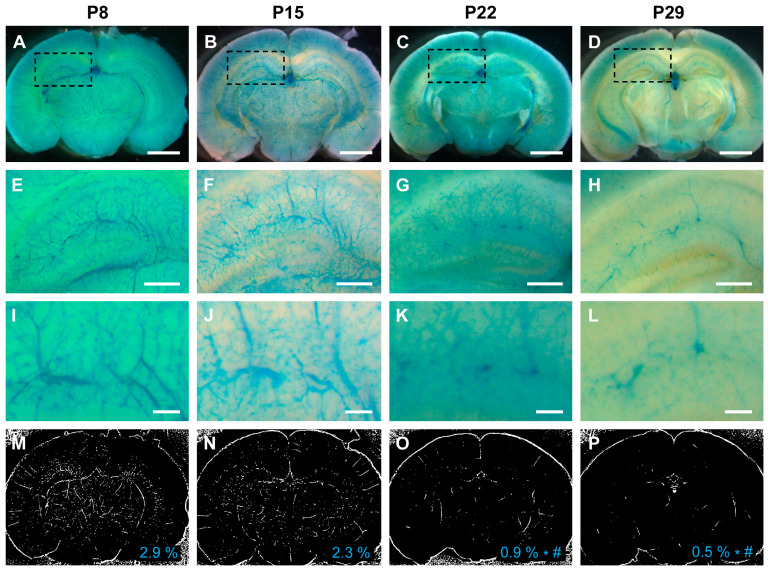
The promoter activity of *Vegfr2* (*Flk1*) in the mouse brain was most pronounced during the first 2 weeks of the postnatal period. (**A**–**D**) The promoter activities of *Vegfr2* are indicated by blue staining within the cerebrovasculature of *Flk1*-LacZ reporter mice at P8, P15, P22, and P29. (**E**–**L**) Dashed rectangular areas in each group indicate the hippocampus. Images of both low-magnification (**E**–**H**) and high-magnification (**I**–**L**) show the promoter activities of *Vegfr2* in the hippocampi at P8, P15, P22, and P29. (**M**–**P**) Representative images illustrate the quantification of *Vegfr2* promoter activities, as measured by the densities of Flk1-positive vessels at P8 (2.9%), P15 (2.3%), P22 (0.9%), and P29 (0.5%). The mean density of each group is displayed in the lower-right corner. The P8 and P15 groups had significantly higher Vegfr2 promoter activities compared with the P22 and P29 groups. *: *p* < 0.001 vs. P8, #: *p* < 0.05 vs. P15. Scale bars: 2 mm (**A**–**D**), 500 μm (**E**–**H**), and 125 μm (**I**–**L**). *n* = 5.

To further validate our results, we leveraged a *Vegfr2* (*Flk1*)-LacZ reporter mouse line, wherein β-galactosidase expression was controlled by the *Vegfr2* promoter. Consistent with our biochemical observations, the activity of the *Vegfr2* promoter in the brain peaked during the first 2 postnatal weeks (2.9% in the first week and 2.3% in the second week), with a significant decline in the third and fourth weeks (0.9% in the third week and 0.5% in the fourth week) (Figure 6).

## 4. Discussion

### 4.1. Postnatal Development of BAVMs in a Type I HHT Mouse Model

In this study, we introduced a novel longitudinal mouse model of familial BAVMs by employing tamoxifen-mediated, endothelial cell deletion of the *Eng* gene during the neonatal stages. Our investigations revealed that BAVMs effectively emerged when *Eng* was deleted within the initial 2 weeks after birth. This discovery signified the contribution of a crucial physiological microenvironment during the early postnatal period, which played a pivotal role in driving BAVM development. The predominant expression of VEGFR2 in the developing mouse brain during this initial 2-week postnatal period strongly suggests the presence of a proangiogenic milieu functioning as an intrinsic co-factor in the development of BAVMs.

### 4.2. The Congenital Nature of BAVMs

The classification of BAVMs as a congenital disease remains a subject of ongoing debate. Previous studies involving HHT mouse models, including both *Alk1* and *Eng* mice, lent support to the hypothesis that BAVMs develop congenitally [17,22,34]. Furthermore, when either the *Alk1* or *Eng* gene was deleted in adult mice, neither BAVMs [22,34] nor skin AVMs [17] developed. However, BAVMs did manifest when VEGF expression was induced in the *Alk1*- or *Eng*-deleted adult mouse brain [22,34]. Likewise, the infliction of a wound or the induction of VEGF in the back skin of *Alk1*-deleted adult mice resulted in the development of skin AVMs at those sites [17,32]. Collectively, these findings suggest that HHT gene deletion alone is insufficient to induce BAVMs in the adult mouse brain. An additional factor, such as wounding, appears to be crucial for the definitive formation of BAVMs. Within the context of our study, we observed that deletion of the *Eng* gene in endothelial cells during the first 2 postnatal weeks is a critical prerequisite for inducing BAVMs, providing further reinforcement of the prevailing theory that BAVMs are inherently congenital in nature.

### 4.3. Angiogenesis as an Endogenous Tertiary Hit after Genetic Second Hit

Since human HHT patients carry heterozygous mutations in HHT genes, haploinsufficiency has been generally accepted as a model of AVM initiation [40,41]. However, recently, Snellings et al. demonstrated that additional somatic mutations are present in the vascular lesion of HHT patients, implying that the loss of heterozygosity may be required for AVM development [42]. In our study, *Eng* mutants supported the concept of loss of heterozygosity as a critical factor in BAVM formation, as only homozygous knockout mice exhibited BAVMs. Intriguingly, our findings indicate that loss of heterozygosity alone is insufficient to trigger AVMs in the brain; it occurs only when the *Eng* gene is deleted during the first 2 weeks of the postnatal period.

What plays such a critical role in the initial 2 weeks of the postnatal period that leads to the formation of BAVMs? If indeed an endogenous triggering event is effective during this period, what characterizes or defines the nature of this event? The concept of angiogenesis has emerged as a prominent factor in the “secondary hit” hypothesis regarding BAVM pathogenesis [19,43].

In the mouse brain, capillaries constitute the predominant vessel type, accounting for over 90% of the total cerebrovascular length [44]. However, at birth, the capillary network is relatively sparse and incomplete compared with the dense capillary network found in the adult mouse brain. During the first few weeks of life, the cortical capillary network of rodents undergoes a dramatic expansion that is primarily driven by VEGF-regulated angiogenesis. This expansion reaches its peak between postnatal days 15 and 25, after which the angiogenic capacity within the capillary network gradually subsides as the capillary density stabilizes [44,45,46,47]. During this phase of stabilization, both pericyte and endothelial cell proliferation rates decline [45]. The findings of our study align with this temporal pattern of angiogenic activity. We observed that BAVMs developed when the *Eng* gene was conditionally deleted at P1–3, P8–10, and P15–17, but not at P22–24. This suggests that mice produce ample endogenous angiogenic stimuli to facilitate BAVM formation up to P15–17, beyond which there is a gradual decline in angiogenic activity, rendering VEGF levels inadequate to serve as a tertiary hit for BAVM formation.

In rodents, postnatal day 10 is approximately equivalent to the developmental stage of a 40-week gestational period in humans [48]. This timing suggests that the events occurring during the first 2 weeks after birth in rodents are relevant to the prenatal and neonatal development of the cerebrovasculature in humans. This alignment may elucidate why BAVMs are predominantly regarded as congenital lesions and why the occurrence of *de novo* BAVMs is relatively rare compared with congenital cases.

### 4.4. Pathogenesis of De Novo BAVMs

The occurrence of *de novo* BAVM formation in the adult human brain, particularly when angiogenesis is not actively occurring, is a rare and challenging phenomenon to understand. Given that angiogenesis alone is unlikely to serve as a direct catalyst for BAVM formation [49], patients with *de novo* BAVMs may have genetic predispositions for BAVM development, such as carrying HHT gene mutations [12,13] or exhibiting somatic mutations in the *KRAS* gene [9].

Over the course of a lifetime, the stabilized cerebrovascular network can be influenced by an array of events capable of modifying angiogenic activity, such as ischemia [2], traumatic brain injury [7], intracerebral hemorrhage [6], seizures [3], intracranial tumors [5], and inflammatory processes caused by bacterial and viral infections [1]. These inciting events, when combined with genetically predisposed or somatically mutated cerebrovascular cells, may collectively contribute to the development of de novo BAVMs by increasing angiogenic activity beyond a certain threshold. Conversely, it has been reported that *Kras* mutations alone have the capacity to induce *de novo* BAVMs in adult mice without the need for an additional stimulus [27,28]. While this may not entirely align with the congenital theory of BAVMs, it is important to note that *Kras* mutant mice exhibited enhanced VEGF-associated angiogenesis [27,28]. Therefore, it remains plausible that angiogenic stimuli still play a role in the formation of *de novo* BAVMs in cases associated with *KRAS* mutations.

### 4.5. A Novel Mouse Model for Forebrain and Hindbrain BAVMs

In HHT patients, the two most common locations of BAVMs were the frontal lobes (43.6%) and the cerebellum (15.4%) [50], while in other BAVMs, BAVMs occurred more commonly in the frontal lobe (18–21%) and temporal lobe (18–19%) [51,52].

In our previous study, we used *Tagln*Cre(+);*Alk1* mutant mice to establish a longitudinal BAVM mouse model [33]. In that model, the predominant site for BAVM development was the parietal lobe, with BAVMs frequently localized along the posterior cerebral artery or within its vascular territory. In contrast, our present study with *Scl*CreER(+);*Eng*^2f/2f^ mutant mice revealed a different pattern of BAVM localization. Here, mice primarily developed cerebellar BAVMs (48%) and forebrain BAVMs (33%). This model offers a valuable resource for studying both forebrain and hindbrain BAVMs in a longitudinal preclinical framework.

## 5. Conclusions

In this study, we established a novel mouse model to investigate the pathogenesis of BAVMs, with a particular focus on forebrain and hindbrain BAVMs, utilizing *Scl*CreER(+);*Eng*^2f/2f^ mice. Through tamoxifen-dependent, endothelial-cell-specific gene deletion, we demonstrated that the deletion of the *Eng* gene within endothelial cells reliably results in the development of BAVMs during the initial 2-week postnatal period. This timeframe corresponds to elevated expression of the VEGFR2 receptor. The temporal correlation between early postnatal BAVM development and heightened angiogenic activity suggests that angiogenesis may contribute to a specific physiological microenvironment critical for initiating BAVM development. Consequently, our investigations support the prevailing belief that BAVMs are derived congenitally and are primarily governed by angiogenesis as an endogenous additional hit during the early postnatal period.

In summary, our study presents a promising preclinical model for advancing the development of novel therapeutic strategies for BAVM treatment. In addition, it contributes to a deeper understanding of the complex mechanisms implicated in BAVM development.

## Data Availability

The data that support the findings of this study are available from the corresponding author upon reasonable request.

## Supplementary Materials

The following supporting information can be downloaded from https://www.mdpi.com/article/10.3390/biomedicines11123153/s1—Figure S1: Survival analysis and hemoglobin studies in the type I HHT mouse model.
