# Supplementary material for: VEGFR2 Expression Correlates with Postnatal Development of Brain Arteriovenous Malformations in a Mouse Model of Type I Hereditary Hemorrhagic Telangiectasia
Source: Biomedicines. 2023 Nov 27;11(12):3153. doi: 10.3390/biomedicines11123153 (PMC10740421; doi:10.3390/biomedicines11123153)
Supplement: Supplementary file 1 [file biomedicines-11-03153-s001.zip › biomedicines-2680715-supplementary.pdf]

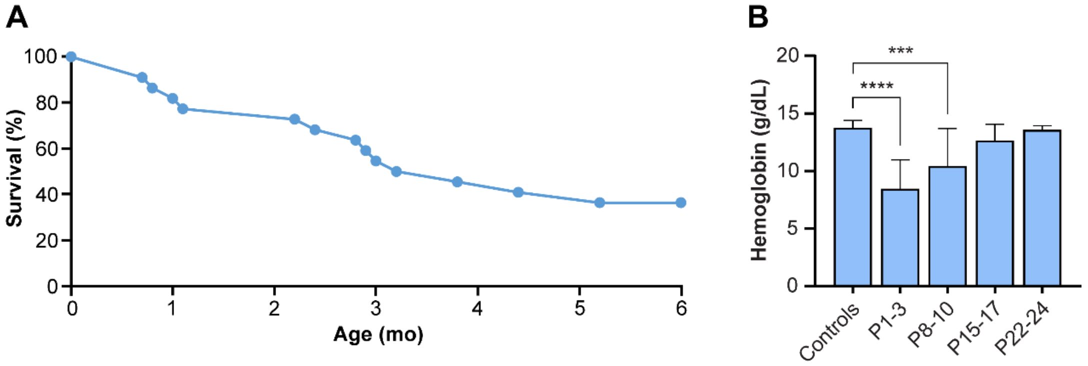

Supplementary Figure S1. Survival analysis and hemoglobin studies in the type I HHT mouse model. **(A)** Approximately 40% of *Eng* mutant mice treated with tamoxifen at postnatal days 1-3 survived up to 6 months of age (N=22). **(B)** The bar graph shows the hemoglobin levels of *Eng*-deficient mutant mice (2-3 months of age) at P1-3 (mean [SD], 8.4 [2.5] g/dL, N=14), P8-10 (10.4 [3.3] g/dL, N=7), P15-17 (12.6 [1.4] g/dL, N=11), P22-24 (13.6 [0.4] g/dL, N=8) groups, and their controls (13.7 [0.7] g/dL, N=17). All tamoxifen-treated, CreER-negative control groups exhibited similar results and were not statistically different from each other. Thus, in the graph, one control group represents combined data from all control groups. The P1-3 and P8-10 groups had significantly lower hemoglobin levels compared to their controls (2-3 months of age). **** $P < 0.0001$ vs controls, *** $P < 0.0008$ vs controls.
